# Experimental and Numerical Assessment of Temperature Field and Analysis of Microstructure and Mechanical Properties of Low Power Laser Annealed Welded Joints

**DOI:** 10.3390/ma11091514

**Published:** 2018-08-23

**Authors:** Uday Kumar, D. K. Gope, J. P. Srivastava, Somnath Chattopadhyaya, A. K. Das, Grzegorz Krolczyk

**Affiliations:** 1 Department of Mechanical Engineering, Indian Institute of Technology (ISM) Dhanbad, Dhanbad 826004, India; kumaruday2404@gmail.com (U.K.); dkrg05@gmail.com (D.K.G.); somuismu@gmail.com (S.C.); eralok@yahoo.co.in (A.K.D.); 2 Department of Mechanical Engineering, S R Engineering College, Warangal, Telangana 506371, India; jaysrvstv@gmail.com; 3 Department of Manufacturing Engineering and Automation, Opole University of Technology, 45-271 Opole, Poland

**Keywords:** laser welding, temperature field, heat affected zone, heat source, weld bead

## Abstract

In this present work, laser welding experiments were carried out on 1 mm thin Ti6Al4V sheets using a low power Nd-YAG laser machine without using any filler wire and without edge preparation of welding specimens. The influence of different major process control parameters such as welding speed and power on the yield parameters like temperature field, weld bead geometry, microstructure, and mechanical properties are critically investigated. Experimental results are compared in detail with the simulated results obtained using a commercial 3D finite element model. In the simulation model, temperature-dependent thermal and mechanical properties of plates were considered. The temperature readings were recorded with the aid of K type thermocouples. Forced convection has been assumed near weld zone region because of the movement of the shielding gas. Appreciable agreement is found between the experimental and the simulated temperature fields in most of the cases with few exceptions. These deviations on few occasions may be due to the presence of uncertainties inherently present in the experimental domain and uncertainties in the subsequent temperature sensing techniques by the thermocouples. In addition, annealing has been done at 950 °C, 980 °C, and 1010 °C for one selected parameter (192 W, 6 mm/s). The tensile strength of the samples annealed at 980 °C has been found to be 1048 MPa and it is 3% to 4% higher than that of the usual welded samples.

## 1. Introduction

In the recent decade, among all the titanium alloys the Ti6Al4V alloy has been extensively used in research work and industrial applications [1,2]. The titanium alloys have comparatively high operating temperature, low density, and good corrosion resistance, and in addition, Ti6Al4V has some more exceptional features like high strength, low thermal conductivity, and chemical reactivity [3]. The fabrication of Ti6Al4V is extremely challenging with conventional machines but laser has solved this problem to a great extent. For this alloy, in most of the cases, both pulsed mode as well as continuous mode laser source can be used. At room temperature Ti6Al4V exists in two phase α and β, in which aluminum stabilizes alpha and vanadium stabilizes beta. Ti6Al4V has higher tensile strength even at temperature 450 °C. The alloy also has low density and high strength. Upon heat treatment, this alloy exhibits excellent mechanical and thermal properties [3]. Laser is a high-density power source that can even weld moderately thick material with good penetration depth in a single pass. This type of welding is very precise and fast in processing. Due to these features, this process has appreciably high efficiency and significantly low cost of production as compared to the other competing welding processes. Another exceptional feature with the laser welding is that it produces less distortion even in long sheets. Akman et al. [4] worked on the welding of thin Ti6Al4V alloy sheet using a high power pulsed Nd-YAG laser machine. The results indicate that by precisely controlling the input parameters it was possible to control the depth of penetration. A meticulous comparative analysis was carried out by Gao et al. [5] between Nd-YAG laser and traditional fusion welding process of thin Ti6Al4V alloy sheet. It was reported that less distortion, low heat affected zone, high microhardness, high strength, high ductility, and fine microstructure of the Nd-YAG laser welded sample as compared with the conventional TIG (Tungsten Inert Gas) welding process.

Way back in 1946 Rosenthal attempted to investigate the temperature field in the welded plate [6]. After that several studies reported on the thermal field which is affected by laser welding parameters. Deng and Murakawa used a 3D, thermo-elastic plastic large deformation FEM (finite element method) to critically investigate the temperature field, distortion, and residual stresses in thin welding plates and experiments were also carried out to do the comparison. The results of the experimental data predominantly matched with that of the simulated data [7]. Again Deng et al. reported the computational approach considering material nonlinearity and geometrical nonlinearity to investigate the distortion and residual stresses. Meanwhile, experiments were also done to carry out comparative assessment [8]. Yilbas et al. studied the thermal and stress field using FEM and welding experiments were done using nitrogen as shielding gas. It was found that the decrease in temperature rate in the molten zone was lower than that of the solid zone [9]. Adamus et al. worked on numerical and experimental analysis of two different grades of titanium for electron beam welding. From the result it was evident that using a welding gap in the model helps predicting transverse bending deformation [10]. Du et al. proposed a mathematical model to investigate the metal flow behavior in full penetration laser welding. Results indicated that the formation of hourglass structure was mainly due to the metal flow [11]. Zhang et al. presented the mechanism of surface underfill in full penetration fiber laser welding of stainless steel. It was shown that the main reason of surface underfill was the downward flow of the molten metal created due to the recoil momentum [12]. Zhang et al. carried out a numerical simulation of full penetration laser welding of 10 mm thick steel plate. During the welding the keyhole behavior and molten flow of both the top and bottom surface were analyzed in detail [13]. Venkatesh et al. studied the influence of heat treatment on the mechanical properties, after welding. Results indicate that the yield strength and ultimate tensile strength of the specimens were higher in water quenching than air cooling [14]. Zhao et al. investigated the effect of post weld heat treatment on high strength steel. It was concluded that with the appropriate heat treatment the ductility increases but strength decreases [15]. Chen et al. also reported the influence of annealing on microstructure and mechanical properties of electron beam welded joints. The microstructure shows that the O particle precipitated out of B_2_ phase and also the size increased in between the temperature 750 °C to 900 °C [16]. 

In the present research work, experimental and numerical analysis of temperature field during laser welding is carried out. A 3D FEM model has been used for the numerical solution. In the boundary condition two modes (convection and radiation) of heat transfer have been considered. In the convection section of the upper surface, two types of convective heat transfer have been used. The forced convective heat transfer for the region near to the HAZ (Heat Affected Zone) and natural convective heat transfer for the rest of the upper surface is considered. Apart from that, annealing of selected samples have been performed at different temperatures to improve the tensile strength.

## 2. Experimental Procedure and Material

### 2.1. Welding Material

In this present work, 1 mm thick Ti6Al4V alloy sheet was used as experimental material. The material composition (wt. %) from the EDX analysis and its mechanical properties have been presented in Table 1 and Table 2, respectively.

### 2.2. Experimental Setup and Procedure

**Figure 1:**
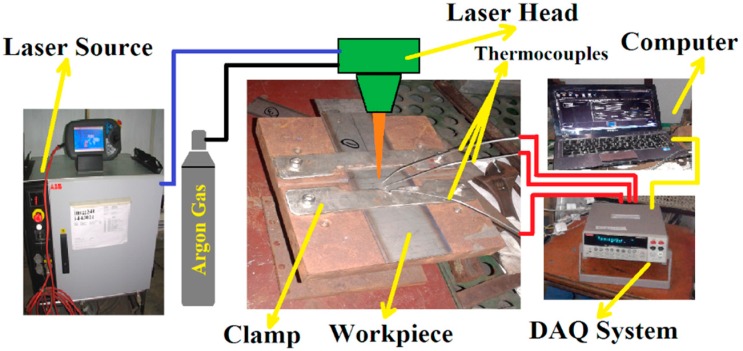
Schematic illustration of complete experimental setup.

A pulsed wave mode 400 W Nd-YAG laser was used to weld 1 mm thick titanium alloy (Ti6Al4V) sheets. Table 3 indicates the constant weld parameters used in this particular work. Argon gas was used as the shielding gas with a flow rate of 8 L/min. The supply of argon gas was only from the top side, which was attached to the laser head. Figure 1 illustrates the entire experimental welding setup with the specimen dimension of 165 × 50 × 1 mm. To investigate weld quality significantly, two major process control parameters—power (P) and the welding speed (V)—were chosen for variation during experimentation. The welding speed was 4–6 mm/s with a defocusing distance of 3 mm during the experimentation. The specimens were placed over a welding bed and just below the laser head. For proper alignment, all four corners of both the thin sheets were kept at right angles. After proper alignment and clamping, the two sheets were welded together. Initially, a few welding tests were conducted to discover suitable values of process parameters to attain good penetration, better surface finish, and visual inspection. In a few cases, the power was kept constant and welding speed was varied, and in other cases, welding speed was kept constant while power was changed. From the several welding tests, four combinations of laser power and welding speeds were selected for obtaining acceptable penetration and appreciable weld quality as shown in Table 4. Lots of welding samples were required for satisfactory evaluation of the different relevant weld properties. For inspection of the samples, polishing was carried out with the standard grinding machine and chemically etched by Keller’s reagents solution (1% HF, 1.5% HCl, 2.5% HNO_3_, and 95% H_2_O) for 20 s. Microstructural analysis was performed by optical microscopy (OM) and field emission scanning electron microscopy (FESEM) to critically investigate the weld samples.

### 2.3. Thermal Measurement

**Figure 2:**
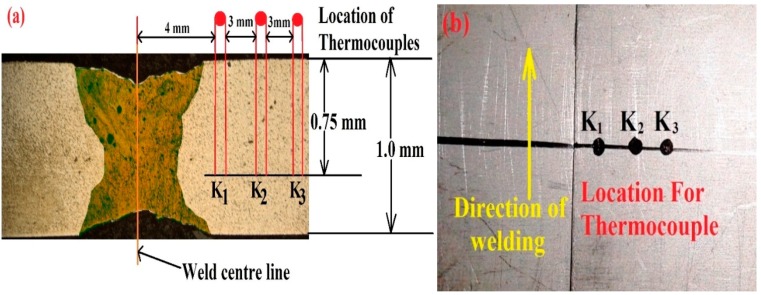
(**a**) Location of the thermocouple from weld center. (**b**) Top view of sample with thermocouple position.

To study the temperature distribution profile at three different locations, three K-type thermocouples were placed perpendicularly as shown in Figure 2. The thermocouples were each 2 mm in diameter and 500 mm long. The first thermocouple was placed 4 mm away from the weld bead center. Second and third thermocouples were mounted at a distance of 3 mm each in the direction as shown in Figure 2a. The measurement range of each thermocouple was −40 to 1200 °C. A computer system and data acquisition system (DAQ) were utilized to acquire the temperature distribution profile. The complete thermocouple arrangement is presented in Figure 2.

### 2.4. Annealing

In order to improve the mechanical properties further, the selected specimens were subjected to three annealing treatments, at 950 °C, 980 °C, and 1010 °C, respectively, for one hour duration. A laboratory heating furnace was used for the annealing process. The maximum heating capacity of the furnace was up to 1200 °C. The inside material of the furnace was refractory bricks and the dimensions were 400 mm × 400 mm × 600 mm. After the heating process, the oven gate was kept closed for one hour and then the sample was taken out. Once again, the heat-treated samples were prepared as indicated above for the analysis of microstructures, tensile test, and microhardness values.

## 3. Numerical Simulation

### 3.1. Mathematical Description of the Model

**Figure 3:**
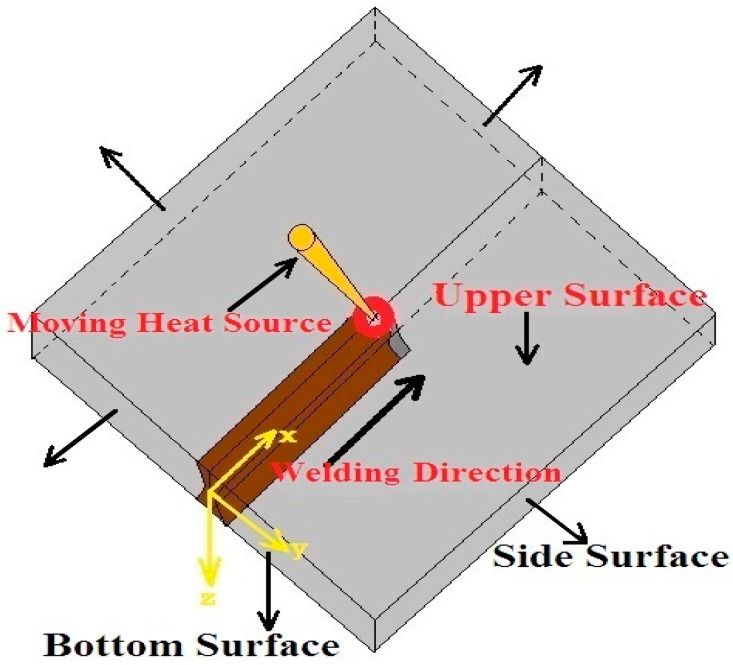
Schematic model for laser welding.

A mathematical model was used to observe the temperature distribution profile during the laser welding process. The nature of heat source distribution was Gaussian distribution. The geometrical configuration of the model has been shown in Figure 3. The 3D transient heat conduction equation for the geometrical model during the welding is given by:

$$\;\left\{\frac{\partial }{\partial x\;}\left[k\left(T\right)\frac{\partial T}{\partial x}\right]+\frac{\partial }{\partial y}\left[k\left(T\right)\frac{\partial T}{\partial y}\right]+\frac{\partial }{\partial z}\left[k\left(T\right)\frac{\partial T}{\partial z}\right]\right\}+Q^{\prime }\left(T,t\right)=\rho c_{p}\left(T\right)\frac{\partial T}{\partial t}+\rho c_{p}\left(T\right)V_{x}\frac{\partial T}{\partial x}$$

where ρ is the density, k is thermal conductivity, T is the temperature, t is time, $c_{p}$ is the specific heat, $Q^{\prime }$ is the rate of internal heat generation, and $V_{x}$ is the laser welding speed. In order to make the calculation a pragmatic one, the model is solved using certain key assumptions.

- Workpiece is moving only in one direction, i.e., *x*-direction.
- Workpiece moving velocity is constant.
- Top surface of the weld pool is considered to be smooth and flat.
- The effect of shielding gas is neglected.
- Half of the geometry is considered to make it less cumbersome.
- Initially the work piece is at a temperature of 300 K (room temperature).
- All the thermophysical properties of the workpiece are considered to be temperature dependent.
- During welding operation, the molten flow is assumed to be incompressible and laminar.
- A 3D time dependent flow is considered for the case.

The initial condition at time t=0 is indicated as $T\left(x,y,z,t\right)=T_{0}$ = 300 K = ambient temperature.

**Figure 4:**
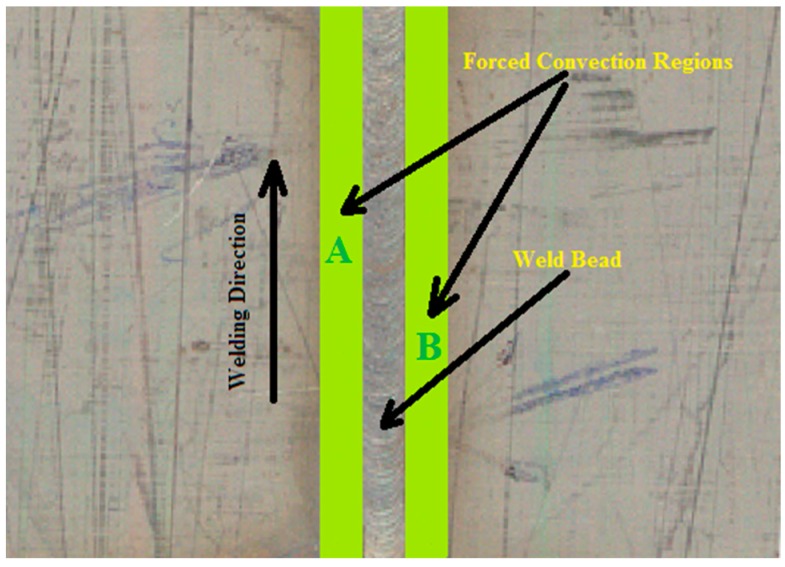
Region A and B indicates the forced convection.

The convection and radiation heat losses were considered at all surfaces. Newton’s law of cooling was used to report the convection heat loss and the radiation heat transfer was represented by Stefan-Boltzmann’s expression. However, in addition to that, the top surface has the laser heat source. Hence, for Z=0 the equation will be

$$\;k(T)\;\frac{\partial T}{\partial z}=q_{L}-\varepsilon \left(T\right)\sigma \left(T^{4}-T_{0}^{4}\right)-h_{avg}\left(T\right)\left(T-T_{0}\right)$$

where$\;h_{avg}$ is the coefficient of convective heat transfer. At the top surface two types of convective heat transfer were considered. The regions Α and Β as shown in Figure 4 were considered for the forced convection because the shielding gas (argon) was passed over it. The rest of the top surface was considered for natural convection through air. In the calculation of forced convection, the fluid properties of argon were considered having a temperature of 27 °C. The natural convective heat transfer coefficient was calculated using the following correlations [17,18]:

$$\left(\begin{array}{c} Nu=\frac{hL\;}{k}\\ Nu=c.{\left[Gr_{L}.Pr\right]}^{n}\\ Pr=\frac{\mu C_{p}}{k}\\ Gr_{L}=\frac{\rho^{2}L^{3}\beta g\left(T-T_{\infty }\right)}{\mu } \end{array}\right)$$

where μ is dynamic viscosity, *L* is the length of region A and B. $\beta =\frac{1}{T}$, *β* is the volumetric thermal expansion coefficient and *T* is absolute temperature of the ideal fluid. ρ = density (4420 kg/m^3^). The value of *c* and *n* depends upon types of flow and position of the plate (*c* = 0.54 and *n* = 1/4) [17]. For the region A and B, the following correlations were used [19]:

$$\left(\begin{array}{c} h=\frac{13\;Re^{1/2\;}Pr^{1/3}k_{Ar}}{l}\\ Re=\frac{V_{Ar}\rho_{Ar}D}{\mu_{Ar}}\\ Pr=\frac{\upsilon_{Ar}}{k_{Ar}} \end{array}\right)$$

where $k_{Ar}$ is the thermal conductivity of argon gas. l is the height between argon nozzle to top surface of the plate, σ is Stefan-Boltzmann constant = 5.67 × 10^−8^ Wm^−2^ K^−1^, *ε* is the emissivity.$\;\varepsilon ={\left\{\frac{T_{r}}{T_{s}}\right\}}^{4}$, where $T_{r}$ is the radiance temperature and $T_{s}$ is the true temperature [20].

$q_{L}$ is the laser heat flux $q_{L}\left(x,y,z,t\right)=\gamma \left(t\right).\frac{2P_{abs}}{\pi r^{2}}e^{-\left(\frac{2x^{2}}{r^{2}}\right)}e^{-\left(\frac{2y^{2}}{r^{2}}\right)}e^{-\left(\frac{2z^{2}}{r^{2}}\right)}$ for *Z =* 0

$$\;q_{L}\left(x,y,t\;\right)=\frac{2P_{abs}}{\pi r^{2}}e^{-\left(\frac{2x^{2}}{r^{2}}\right)}e^{-\left(\frac{2y^{2}}{r^{2}}\right)}$$

$$\;P_{abs\;}=\eta P_{inc}$$

where $P_{T}$ is the total intensity of the laser beam, *r* = radius of laser beam, $P_{abs}$ = total absorbed power, $P_{inc}$ = total incident power, η = average absorptivity of the material. η(Ti6Al4V) = 0.34 [21].

### 3.2. Finite Element Model

**Figure 5:**
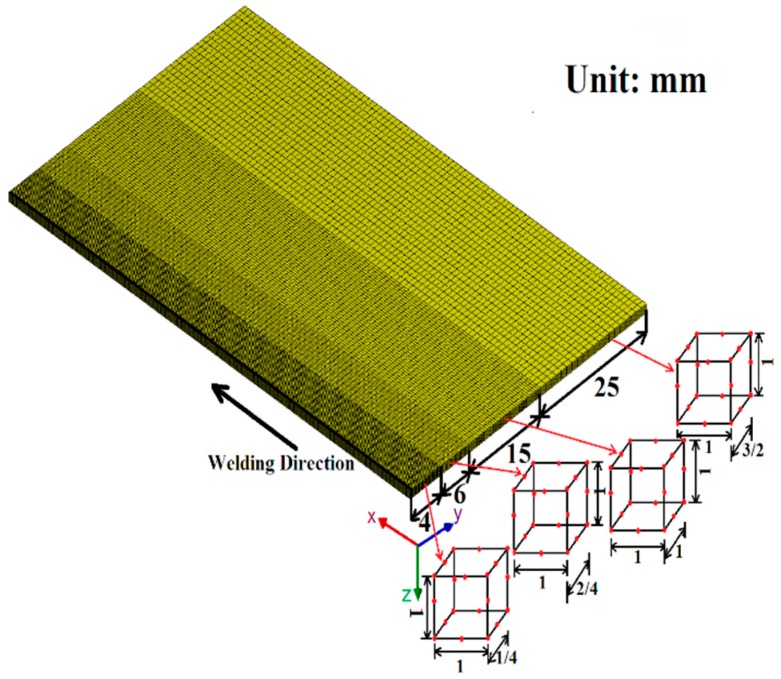
Description of meshes used in the simulation.

A 3D FEM is used to analyze the temperature distribution induced during laser welding. The model was simulated using ANSYS software. Figure 5 represents the meshing pattern used in simulation. For transient thermal analyses, the simulation domain is meshed with 20 noded SOLID 90 element. The finite element model includes 44,225 elements and 81,304 nodes. A refined mesh is used near the weld zone to accurately capture the temperature field. The element size is made gradually coarser moving away from the fusion zone to reduce the computational effort. The simulation is done only for half of the geometry due to its symmetric nature.

## 4. Result and Discussion

### 4.1. Thermal Field

**Figure 6:**
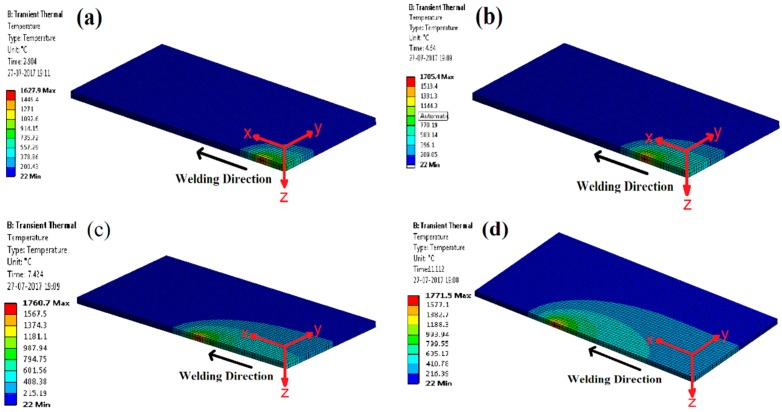
Transient temperature distribution at different time steps: (**a**) t = 2.904 s; (**b**) t = 4.64 s; (**c**) t = 7.424 s; (**d**) t = 11.112 s.

Figure 6a–d represent the temperature distribution profile at different time intervals during the welding simulation of the weld plate. The temperature profile includes the FZ (Fusion Zone), HAZ, and BM (Base Metal) in all cases. Figure 6a shows the temperature distribution after 2.904 s from the start of the welding and maximum temperature reaches up to 1628 °C. Similarly, Figure 6b–d show the temperature distribution after 4.64 s, 7.424 s, and 11.112 s, respectively, from the beginning of the welding and maximum temperature reaches up to 1705 °C, 1761 °C, and 1771 °C, respectively. From the images it is evident that the decrease in temperature as distance increases from the weld line along the transverse direction, has a non-uniform pattern. The main reasons are the uneven cooling and different thermal properties of material at different temperatures [22].

**Figure 7:**
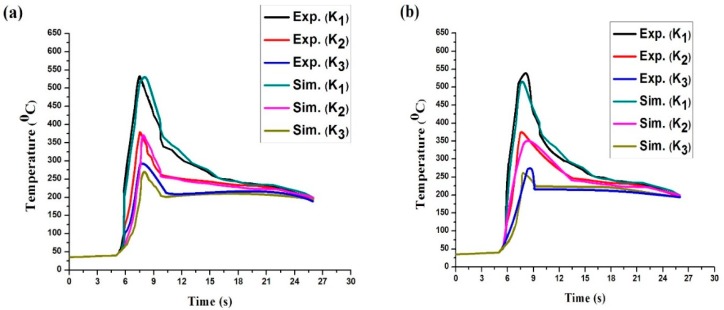

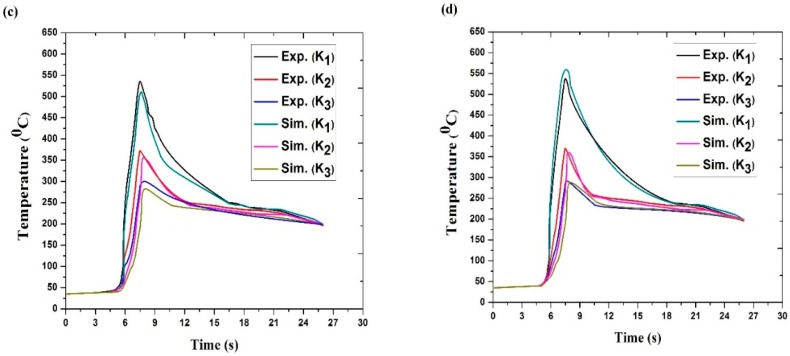
Experimental and numerical result for thermal histories at different parameters: (**a**) 192 W and 4 m/s; (**b**) 192 W and 6 m/s; (**c**) 202 W and 4 m/s; (**d**) 202 W and 6 m/s.

Figure 7 illustrates the experimental and simulated temperature distribution for the three measurement positions (thermocouples K_1_, K_2_, and K_3_) of the weldments. Figure 7a–d show the thermal histories for the parameters 192 W and 4 m/s, 192 W and 6 m/s, 202 W and 4 m/s, and 202 W and 6 m/s, respectively. In all the cases from the figure, it is evident that the temperature of the element rises rapidly and very soon approaches the melting point temperature level of the material. The temperature of the weld element starts decreasing rapidly, as the heat source moves away. The experimental peak temperature for the thermocouple K_1_ is observed to be maximum in sample b among all samples and it is 541 °C. Similarly, for the thermocouple K_2_ the maximum temperature is 380 °C for sample b, and for thermocouple K_3_ the maximum temperature is 302 °C for sample c. From the graph, it is quite evident that the shape of the thermal histories of both experimental and simulated are almost similar. Hence, the simulated data is in appreciable agreement with the experimental data with some stray deviations here and there due to the existence of experimental noise element.

### 4.2. Weld Bead Analysis

**Figure 8:**
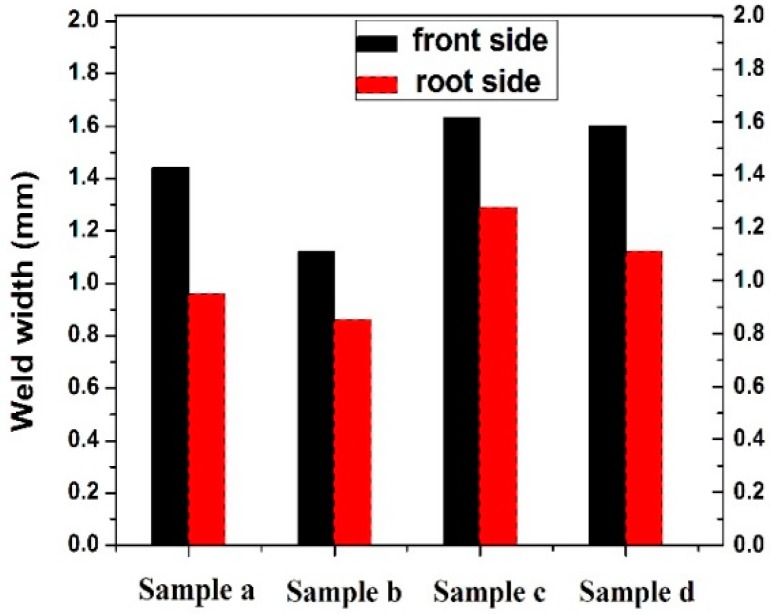
Bar chart of weld width of the different specimens.

Figure 8 represents the bar chart of the weld bead width for the top side as well as root side for different parameters. The larger weld width on the top of the weldment as compared to the bottom part of the weldment indicates the effect of heat input or laser power on the size of weld width. The weld bead width of top surface of samples a, b, c, and d are 1.44 mm, 1.12 mm, 1.63 mm, and 1.60 mm, respectively. The weld bead width of the root side surface of samples a, b, c, and d are 0.96 mm, 0.86 mm, 1.29 mm, and 1.12 mm, respectively. From the result it is clear that on increasing the welding speed, the width of the weld bead on the top side, as well as the root side, decreases. For 192 W, the weld bead width is lower on both sides compared to that of 202 W.

High heat input directly influences the weld bead width and weld bead width plays a vital role in deciding the tensile strength [23]. Here the minimum weld bead is for sample b on both sides of the weld. The maximum weld bead is found in the case of sample c.

### 4.3. Tensile Properties Analysis

**Figure 9:**
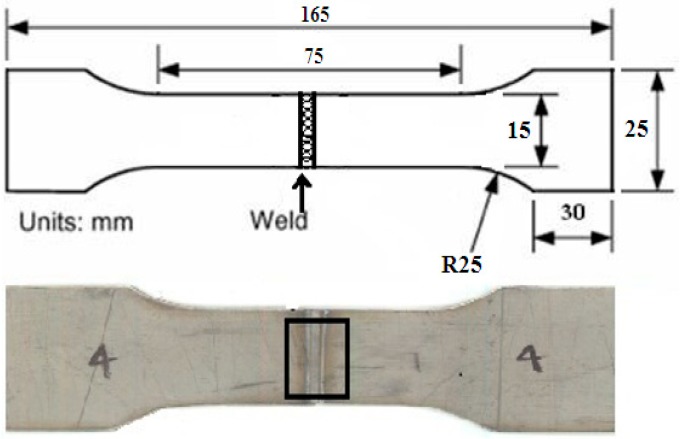
Tensile weld specimen dimension.

**Figure 10:**
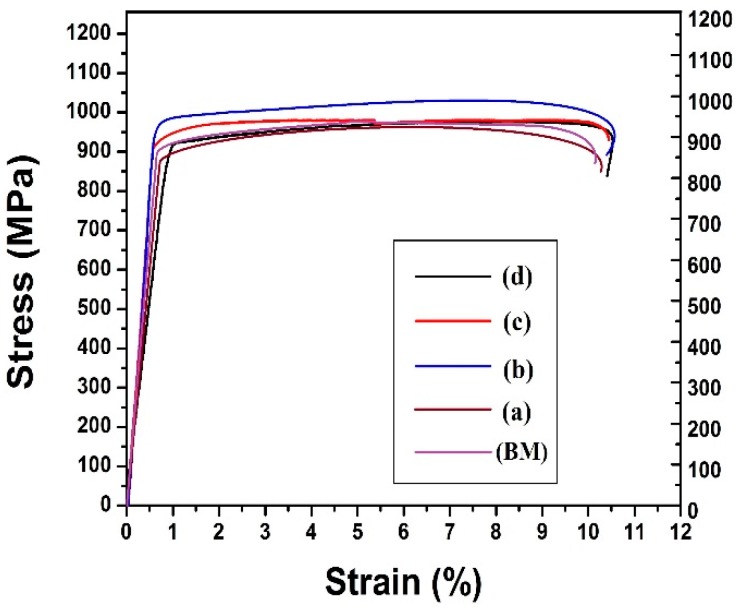
Stress strain curve of the welded joints at different parameters.

For the tensile test the welded specimens were prepared as per the given ASTM E-8 standard [24]. Figure 9 presents the tensile sample along with the dimensions. For this particular work a fully automated close loop servo mechanical tensile testing machine [BISS] was used. The maximum load capacity of this machine was 25 kN. All the tensile tests were accomplished at room temperature (30 °C). The rate of deformation of the tensile test samples was 0.0001/s. An axial extensometer (12.5-mm gauge length) was attached with the help of rubber band at the middle of each specimen for measuring the extension. The data of all the tensile test specimens were recorded by a computer system. Initially, many welded samples and base metal were tested for each parameter. The average value of all the data has been considered for plotting the result. Figure 10 represents the stress strain curve of the base metal and the welded samples. From the graph it can be concluded that the ultimate tensile strength of sample b is higher than that of the base metal and other welded samples. The graph of the base metal and the welded samples overlap each other except for sample b. The ultimate tensile strength of sample b was 1014 MPa and it is 5–6% more than the base metal. In case of sample b, the power was 192 W and welding speed was 6 mm/s. This indicates that the heat required for fusion is sufficient in comparison to the other heat inputs. It emphasizes the fact that the grain refinement of this sample is significantly better than that of the other welded samples.

### 4.4. Effects of Annealing on Microstructure and Mechanical Properties

#### 4.4.1. Microstructure

**Figure 11:**
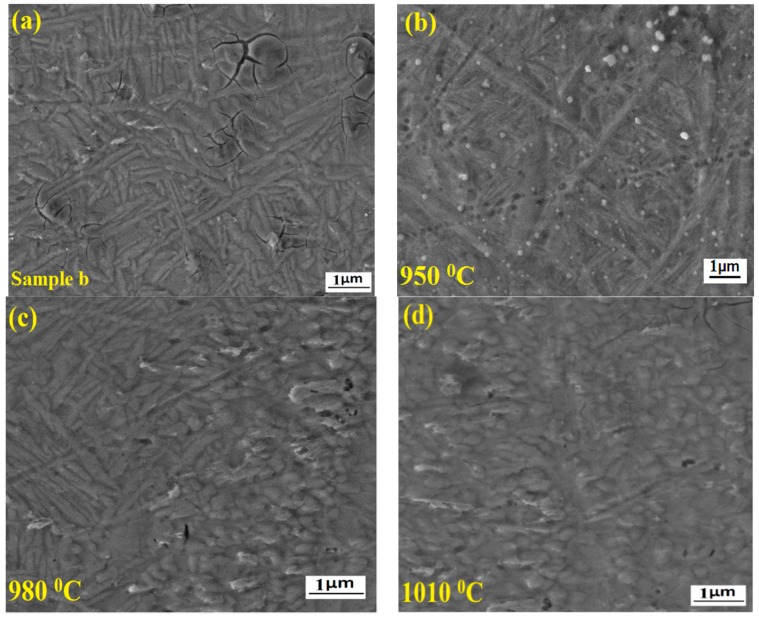
Microstructure of weld joints under different annealing conditions. (**a**) Sample b; (**b**) at 950 °C; (**c**) at 980 °C; (**d**) at 1010 °C.

Figure 11 shows the field emission scanning electron microscopic images of the weld joints annealed at different temperatures. In Figure 11a it can be observed that the microstructure of the fusion zone is full of martensite α’ of needle shape structure with a few cracks. Figure 11b represents the microstructure of the FZ annealed at 950 °C. In this figure it is clear that the martensite α’ needle shaped phase is present but the formation of new grains has been initiated. The cracks have almost disappeared in this figure. This is definitely due to the homogeneous cooling rate. Figure 11c shows the microstructure of the FZ annealed at 980 °C. Here, the new grains have undergone complete transformation into equiaxed α grains. The formation of equiaxed α grains was due to the recrystallization and boundary splitting. Generally, the splitting of boundary occurs due to extensive recovery of the sub boundaries during the heat treatment process [25]. After annealing at 980 °C the specimen shows fully recrystallized microstructure. Hence, in this case the structure is very fine and mostly defect free. Figure 11d indicates the microstructure of the FZ annealed at 1010 °C. As shown in this figure, almost the entire area is full of equiaxed α grains and the grain is coarse in nature in comparison to the previous structure. It means that on increasing the annealing temperature above 980 °C the size of the grain increases progressively.

#### 4.4.2. Tensile Properties Analysis

**Figure 12:**
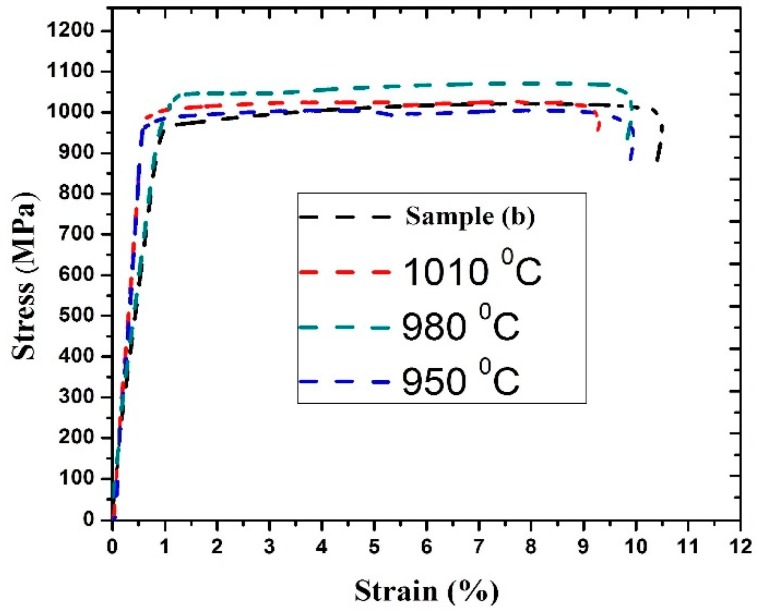
Stress strain curve of joints annealed at different temperatures.

Figure 12 represents the stress strain curve of sample b and annealed samples at 950 °C, 980 °C, and 1010 °C. The ultimate tensile strength (UTS) and elongation value of different samples are listed in Table 5. It indicates that the ultimate tensile strength of sample b and the sample annealed at 950 °C is quite similar. The maximum value of ultimate tensile strength was observed for the sample annealed at 980 °C whereas sample b shows the minimum value of the ultimate tensile strength. In case of elongation (%), sample b exhibits the maximum value and the sample annealed at 1010 °C shows the minimum value. It shows that as the annealing temperature increases, the elongation decreases.

At 980 °C the ultimate tensile strength is maximum, and it is obviously due to the complete grain refinement of the equiaxed α grains. The microstructural inhomogeneity is very low at this temperature in comparison to others. Moreover, the coarse grains are mostly responsible for the decrease in the ultimate tensile strength of the sample at 1010 °C.

#### 4.4.3. Microhardness

**Figure 13:**
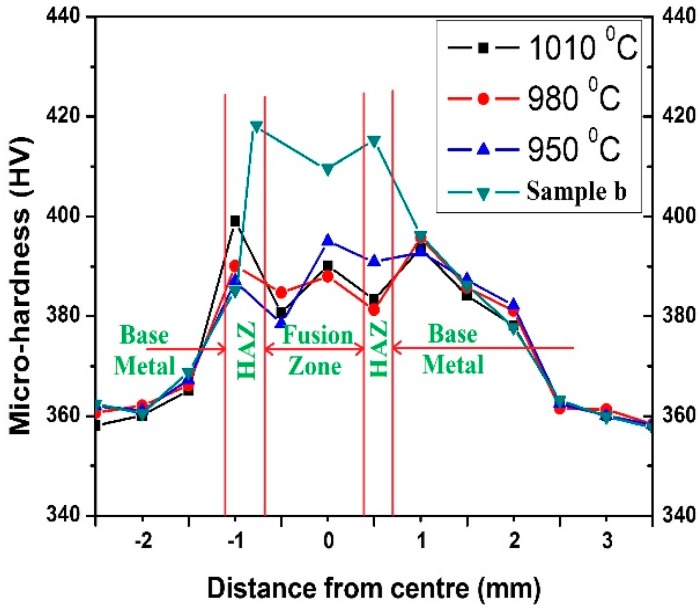
Microhardness of welded joints at different annealed temperatures.

The microhardness distribution of sample b and annealed samples at different temperatures is shown in Figure 13. From the graph it is evident that the microhardness is higher for sample b among all the samples. In sample b, the HAZ reported the highest microhardness value of HV 417 and FZ has a lower hardness value of HV 409. Among the annealed samples the microhardness variation is limited. The hardness value of 1010 °C in the HAZ is highest, HV 399, and it is lowest (HV 381) for 980 °C. The variation of microhardness for the 980 °C is mostly uniform, its hardness value ranges from HV 381 to HV 390. The grain refinement is the main reason for the uniformity in this sample. On the other hand, the coarsening of the grains decreases the hardness value to a great extent. The hardness values are lower at the FZ in the case of the sample annealed at 1010 °C as a result.

## 5. Conclusions

In this paper, numerical and experimental assessments of thermal field of 1 mm thin Ti6Al4V alloy welded sheet were done using low power Nd-YAG pulsed laser machine. For the simulation a 3D finite element model was used. Apart from this, heat treatment has been done on selected samples to improve the tensile properties and microhardness. Based on the findings it can be concluded that:

- An appreciable agreement was found when the simulated results are compared with the experimental results.
- During comparison, the shape of the thermal histories for some parameters were very similar for both simulated and experimental data.
- The microstructures of the weld zone of annealed samples changed to a great extent after annealing. With an increase in annealing temperature, the number of equiaxed α grains increases, and it was maximum for the sample annealed at 980 °C.
- The annealed samples at 980 °C have shown the best tensile strength among all the heat-treated samples.
- The tensile strength of the annealed sample b at 980 °C was observed to be maximum with a value of 1048 MPa and it is 3% to 4% more than that of a conventional weld sample and 8% to 9% greater than that of the base metal.

## Figures and Tables

**Table 1:** Chemical composition (wt. %) of as-received Ti6Al4V alloy.

C	Al	V	O	Ti
3.78	7.08	3.38	0.25	85.51

**Table 2:** Tensile properties and hardness of as-received Ti6Al4V alloy.

Tensile Strength (MPa)	Yield Strength (MPa)	Elongation (%)	Hardness (HV)
964 ± 12	891 ± 19	14	356

**Table 3:** Constant weld parameters used in present study.

Wavelength	1.06 μm
Pulse energy	20 J
Peak pulse power	1000 W
Pulse duration	20 ms
Pulse frequency	20 Hz
Spot diameter	0.45 mm

**Table 4:** Welding parameters used in present studies.

Sample	Average Power (Watt)	Welding Speed (mm/s)
a	192	4
b	192	6
c	202	4
d	202	6

**Table 5:** Tensile properties of sample b and annealed sample at different temperature.

Tensile Properties	Sample b	At 950 °C	At 980 °C	At 1010 °C
UTS (MPa)	1014	1016	1048	1021
Elongation (%)	10.5	9.9	9.7	9.3
